# Supplementary material for: Practical strategies for SARS-CoV-2 RT-PCR testing in resource-constrained settings
Source: medRxiv. 2021 Mar 4:2021.02.18.21251999. Originally published 2021 Feb 25. Preprint. [Version 2] doi: 10.1101/2021.02.18.21251999 (PMC7924294; doi:10.1101/2021.02.18.21251999)
Supplement: Supplement 1 [file media-1.pdf]

Supplementary Material

Table S1: Results of RT-qPCRs across nCoVPC plasmid control concentrations

Target descriptive	Level concentrations (viral copies/uL)	Number of Positive Hit Counts	Total Test Counts
N1	100,000	26	26
N1	50,000	3	3
N1	20,000	33	33
N1	2,000	33	33
N1	200	33	33
N1	20	33	33
N1	10	20	21
N1	2	11	33
N2	100,000	26	26
N2	50,000	3	3
N2	20,000	33	33
N2	2,000	33	33
N2	200	33	33
N2	20	33	33
N2	10	18	21
N2	2	17	33

Table S2: qRT-PCR results for pooled 3D-NMT swabs and concurrently collected individual flocked NMT swabs. Pools, and their comprising individuals, are denoted by shaded rows. Individuals of the household who were not available for pooling are marked as not applicable. 24 pools and 106 individual swabs were collected.

Family ID	Subject ID	Ct value	Avg viral RNA copies/uL (NMT)	Pool result +/-	Ct Value of Pool	Average viral RNA copies/uL of pool
COV004 D21	COV004	undet	0	-	undet	0
	COV004-001	undet	0			
	COV004-002	undet	0			
	COV004-004	undet	0			
COV012 D1	COV012	29	2,439	+	23	54,917
	COV012-001	27	7,196			
	COV012-002	21.5	144,769			
	COV012-003	26	8,223			
	COV012-004	23.5	55,478			
COV017 D1	COV017	28	3,603	+	23	99,275
	COV017-001	23	135,846			
	COV017-002	30	518			
	COV017-003	22.5	162,938			
	COV017-004	21.5	481,149			
COV020 D22	COV020	undet	0	indet	38.5	4
	COV020-001	undet	0			
	COV020-002	undet	0			
	COV020-003	undet	0			
	COV020-004	39.5	3			
COV021 D14	COV021	undet	0	-	undet	0
	COV021-001	undet	0			
	COV021-002	undet	0			
	COV021-003	undet	0			
	COV021-004	undet	0			
COV021 D21	COV021	undet	0	-	undet	0
	COV021-001	undet	0			
	COV021-002	undet	0			
	COV021-003	undet	0			
	COV021-004	undet	0			
COV026 D7	COV026	undet	0	-	undet	0
	COV026-001	undet	0			
	COV026-002	undet	0			
	COV026-003	undet	0			
	COV026-004	undet	0			
COV026 D14	COV026	undet	0	-	undet	0
	COV026-001	undet	0			
	COV026-002	undet	0			
	COV026-003	undet	0			
	COV026-004	undet	0			
COV027 D7	COV027	31	298	+	33.5	34
	COV027-001	35	24			
	COV027-002	35	21			
	COV027-003	35.5	16			
COV027 D14	COV027	undet	0	indet	36	7
	COV027-001	36.5	7			
	COV027-002	33.5	31			
	COV027-003	undet	0			
COV029 D8	COV029	undet	0	+	31.5	131
	COV029-001	38	5			
	COV029-002	35	29			
	COV029-003	22	134,705			
COV030 D8	COV030	29	1532	+	28.5	2183
	COV030-001	undet	0			
	COV030-002	28	2767			
	COV030-003	37.5	5			
COV030 D22	COV030	undet	0	indet	37	3
	COV030-001	undet	0			
	COV030-002	undet	0			
	COV030-003	undet	0			
COV032 D7	COV032	32.5	66	-	undet	0
	COV032-001	undet	0			
	COV032-002	undet	0			
	COV032-003	undet	0			
COV032 D21	COV032	34	24	-	undet	0
	COV032-001	undet	0			
	COV032-002	undet	0			
	COV032-003	undet	0			
COV033 D7	COV033	undet	0	-	undet	0
	COV033-001	undet	0			
	COV033-002	undet	0			
	COV033-003	undet	0			
	COV033-004	undet	0			
COV041 D7	COV041	undet	0	+	24	28,967
	COV041-001	20	371,312			
	COV041-002	36.5	4			
	COV041-003	undet	0			
	COV041-004	undet	0			
	COV041-005	undet	0			
COV045 D7	COV045	undet	0	-	undet	0
	COV045-001	undet	0			
	COV045-002	undet	0			
	COV045-003	n/a	n/a			
COV049 D7	COV049	32	607	+	22	9,468
	COV049-001	27.5	59,704			
	COV049-002	27.5	19,719			
	COV049-003	24.5	892,519			
COV051 D9	COV051	undet	0	-	undet	0
	COV051-001	undet	0			
	COV051-002	undet	0			
	COV051-003	undet	0			
	COV051-004	undet	0			
COV052 D1	COV052	21	270,034	+	21.5	152,869
	COV052-001	undet	0			
	COV052-002	undet	0			
	COV052-003	34.5	22			
COV052 D8	COV052	33	69	+	34.5	14
	COV052-001	undet	0			
	COV052-002	undet	0			
	COV052-003	undet	0			
COV055 D8	COV055	undet	0	+	27	6,152
	COV055-001	37.5	9			
	COV055-002	28	3,800			
	COV055-005	n/a	n/a			
COV059 D8	COV059	undet	0	+	27	6,417
	COV059-001	27	8,367			
	COV059-002	undet	0			
	COV059-003	undet	0			
	COV059-004	undet	0			

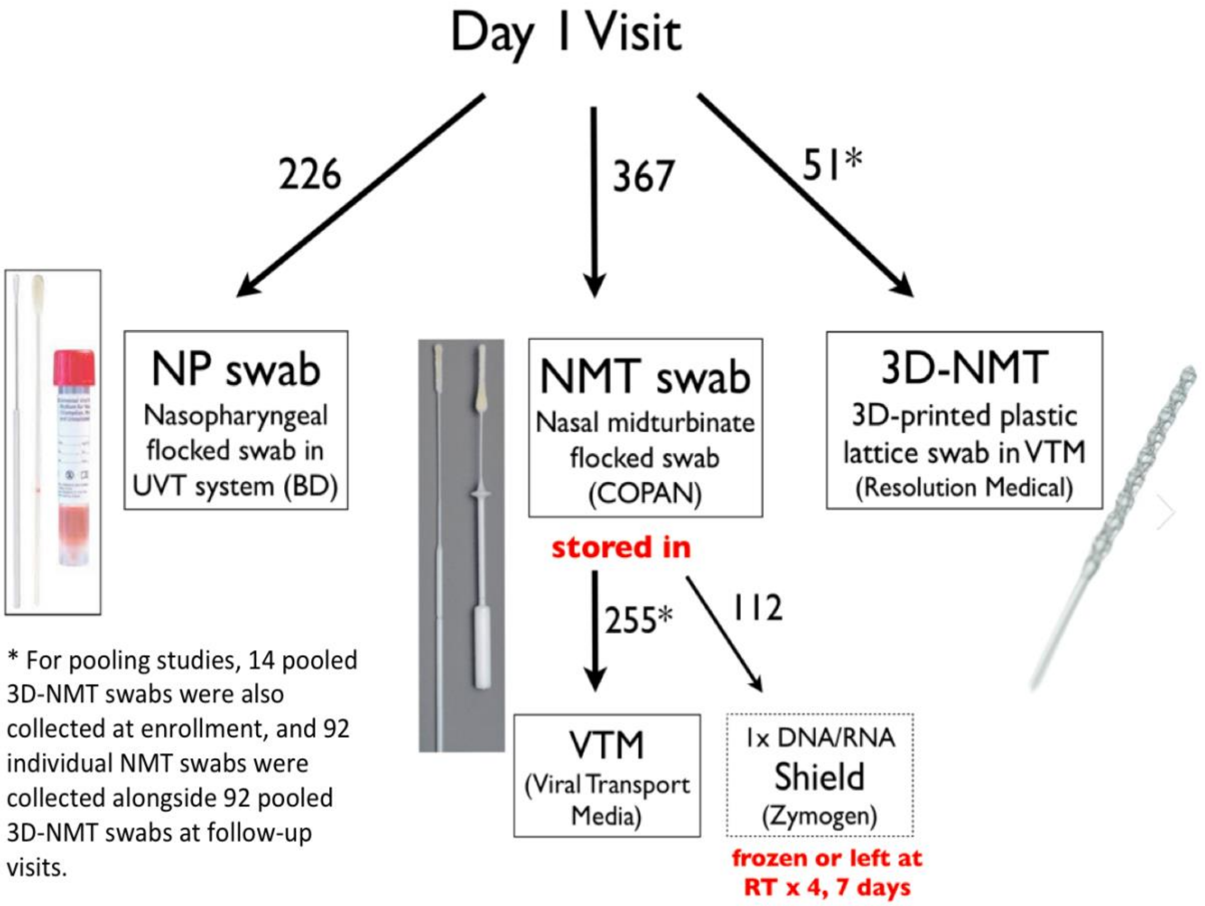

Figure S1. Schematic of swab collection strategies at enrollment. For each study participant at enrollment, a nasopharyngeal (NP) swab was collected as the gold standard diagnostic test. Up to 2 nasal midturbinate swabs - flocked COPAN swabs (NMT) and/or 3D-printed plastic lattice swabs (3D-NMT) - were concurrently collected, one from each nostril, for comparison. 3D-printed swabs were autoclaved before use per manufacturer's instructions

Swabs were either stored in viral transport media (VTM) or 1x DNA/RNA Shield (1xShield). Samples stored in 1xShield were further analyzed via aliquots that were either immediately frozen (D1 Shield) or left out at room temperature for 4 or 7 days (D4 Shield, D7 Shield) before storage at -80°C. A total of 620 individual swabs were collected at enrollment, while 184 swabs were collected to test pooling at follow-up visits.

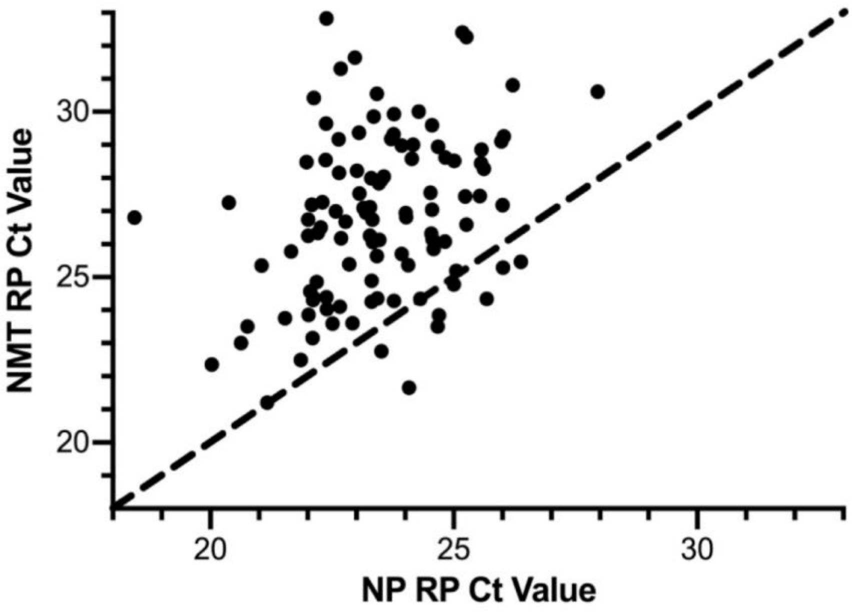

Figure S2. Comparison of human RPP30 gene (RP) cycle threshold (Ct) values from paired nasopharyngeal (NP) and nasal mid-turbinate (NMT) swabs. Higher RP Ct values were observed in NMT swabs compared to NP swabs with an average Ct value difference of 3.1 cycles, suggesting better sampling. A y=x line is drawn for reference.
